# Engaging Transgender Patients: Using Social Media to Inform Medical Practice and Research in Transgender Health

**DOI:** 10.1089/trgh.2017.0039

**Published:** 2018-12-26

**Authors:** Charlie Blotner, Micah Rajunov

**Affiliations:** ^1^ School of Social Work, The University of Washington, Seattle, Washington.; ^2^ Questrom School of Business, Boston University, Boston, Massachusetts.

**Keywords:** transgender, barriers to care, community participatory research, social media, medical education

## Abstract

Despite accelerated growth in the field of transgender health, mistrust between trans patients and their providers persists. Insufficient education for providers, research that overlooks patients' foremost concerns, and continued stigma and discrimination in medical settings are among the main barriers leading trans patients to delay or avoid care. Consequently, the transgender community often turns to social media as a self-serve resource for medical knowledge. This article discusses the benefits of directly engaging the trans community using social media to educate professionals, drive relevant research, and inform practice, to ultimately deliver higher quality care.

## Introduction

The transgender community faces many barriers to care; among these are inadequate provider training, lack of information that reflects patients' expressed needs, and discrimination in medical settings.^1–3^ For decades, trans patients have been filling in the gaps of their own care, often out of fear and distrust of medical professionals.^4^ Instead of relying on providers, patients frequently look inwards toward their community for medical knowledge and advice. Social media, in particular, enables this self-serve model to thrive.^5,6^ To improve the quality of trans health care, providers and researchers must not only expand our body of medical knowledge but must also work to reduce barriers to care. In this article, we discuss how providers and researchers can begin addressing these specific barriers by engaging directly with trans voices across all health care touchpoints. Social media provides a useful tool through which the medical community can connect with transgender patients to learn firsthand about their real needs and experiences, subsequently informing practice, education, and research.

## Barriers to Care in Transgender Health

Lack of trans-specific expertise has been found to be one of the largest barriers to care, yet the necessary medical curricula are largely absent.^2,7^ A median of five hours is dedicated to teaching LGBT-related content, and a third of programs reportedly spend 0 h on LGBT health-related content during clinical training.^8^ Three quarters of medical students report receiving less than two hours of curricular time devoted specifically to transgender clinical education.^9^ This oversight in education extends to practice; while resources do exist, providers are often at a loss for how to fill in their knowledge gaps.^10^ In a 2017 study of practicing endocrinologists, 80.6% reported no training on the care of transgender patients, although 80% had treated a trans patient.^11^ In another recent study, nurse practitioners expressed their frustration at the lack of published evidence to guide practice, turning to the media or their patients for information.^12^

Despite an increased availability of research in trans health, the answers (and even the questions) that matter *most* to patients are notably absent from the medical literature. For example, over half (51.5%) of trans masculine individuals bind their chests daily; however, there have been only *two* studies on chest binding to date.*^,13^ Similarly, while only one in four trans people access transition-related surgery, a disproportionate amount of clinical research is devoted to surgery.^14^ The questions raised on social media point to ways in which trans patients are not receiving proper information about their care, as well as felt needs that are being overlooked by current research.^6,15^

The result is a community hesitant to interact with the medical system. Nearly one-fifth of transgender patients delay health care because of fear of discrimination.^14,16^ Inappropriate provider behavior, such as purposely misgendering, asking unnecessary anatomical questions, and gossiping or mocking, further dissuade trans patients from seeking care.^3,17^ Patients of color and/or with disabilities experience higher levels of discrimination, in addition to those facing transportation, financial, or insurance barriers that prevent them from accessing care in the first place.^3,7,14,18,19^

A first step toward identifying and addressing these gaps in care is to establish conversations that bridge disparate stakeholders with a shared interest in transgender health. Yet even when invited to do so, trans patients may be hesitant to educate medical providers or participate in research.^20^ Concerns around logistics, fear of being outed, and minimal compensation, along with a lack of awareness of participant opportunities, all contribute to this friction.^4,7^ Thus, increasing trust between trans patients and providers is a critical element for improving communication between these parties.

## Using Social Media to Engage Trans Voices

Other medical fields have recognized the benefits of using online spaces to build trust with patient communities, in part, for the unprecedented access to patient conversations. For example, the field of oncology found that interactions on social media transcended patient-provider hierarchies and facilitated meaningful education.^21^ Engagement through social media can generate peer-to-peer education and foster support, and debunk health myths.^22^ Learning about patients outside of clinical settings allows professionals to see their patients as individuals rather than stereotypes, people to fear, medical oddities, or merely “teachable moments.”^23^

The transgender community thrives on social media as a resource and path to authenticity, permitting the negotiation of what it means to have a marginalized identity, while allowing for anonymity when needed.^5,24^ Medical professionals can engage with the trans community on an equal plane through online forums (Instagram, YouTube, Facebook, Reddit, and Twitter), where popular trans individuals boast 50,000, 100,000, and even upwards of half a million followers. Social media serves as a platform to discover resources, learn about gender-affirming providers, and build community in the process.^5,6^ A recent study by Seattle Children's Hospital found that the Internet plays a particularly strong role in the lives of trans youth and their caregivers.^15^ Another instance of successful patient-provider interaction online can be seen in Reddit's Transgender Health AMA thread.^†^ Active participants included prominent trans health providers and institutions^‡^ such as Dr. Johanna Olson-Kennedy, Dr. Joshua Safer, and Fenway Health; they answered questions posed directly by patients, sharing evidence-based information with the community.

The authors created an online medical perspectives series, *Transgender Health: Patients and Providers,*^§^ as another model for promoting patient-centered understanding of trans needs and trans health. Since its launch, over 100,000 views and almost 50,000 unique visitors have found their way to *Patients & Providers*. The series features guest articles from international providers, representing the fields of psychology, endocrinology, pediatrics, emergency medicine, surgery, social work, nursing, and research, in addition to patient perspectives. By offering themselves as resources, both parties reduce the friction for interactive medical education. The series is hosted on a website** recognized for its accessible intercommunity knowledge, with easy to understand resource articles about nonbinary identity and transition. Medical professionals also make use of this platform, and it is referenced within the UCSF Center of Excellence for Transgender Health Guidelines.^††^ This dual use by both patients and providers makes it an ideal online space to invite providers into conversations they are already listening to, but perhaps not actively participating in. In one popular article,^‡‡^ questions were sourced directly from trans community members through social media and reader e-mails, with answers written by primary care physician Dr. Kevin Hatfield. This post-exemplifies active provider engagement with patients' foremost questions and concerns.

However, the limitations to social media must be noted. Anecdotally, some providers reached out to the authors privately (by e-mail or Twitter Direct Message); they were hesitant to publicly endorse practices that are not yet fully supported by medical associations (e.g., starting/stopping hormones and informed consent), even though they use these in their medical practice. While patients are afforded the safety of online anonymity, providers may feel compelled to self-identify, to affirm their legitimacy as professionals. As one commenter shared, “Currently, there's silence and shame on both sides of the Dr.'s table.”^§§^ Among the technical shortcomings, it can be difficult to quantify the impact of this and other online efforts. Real-time or elaborate back and forth dialogue can be stunted on platforms with character limitations (Twitter), nonthreaded posts (Tumblr), or partial digital privacy (Facebook). Moreover, online samples may skew toward a representation of younger, genderqueer participants; they are not a reflection of all trans identities or needs.^25^

## Conclusion

Our ability to deliver optimal trans-specific health care is severely limited by insufficient trans-specific medical education, a paucity of research that addresses the expressed needs of patients, and discriminatory behaviors in medical settings. Providers guessing what their patients want not only fails to help but can also potentially cause harm. Trans patients need to feel safe enough to seek out trained medical professionals informed by relevant evidence-based research and affirming practices.

The authors recommend using social media to drive professionals' exposure to, and understanding of, transgender lived experiences. Specific engagement tactics can include following blogs and individual accounts, or participating in virtual conversations through community hashtags, to track the diffusion of inaccurate health information, demystify frequently asked questions, or inform crucial talking points overlooked in patient interaction. Virtual interactions also mitigate some of the institutional, logistical, and social barriers that prevent the transgender community from trusting local and global providers in person. In addition, using these transparent data to inform practice and research questions holds institutions and researchers accountable to a public, community audience. Trans-led initiatives on popular platforms bring together both patients and providers as experts of their experience, inviting us all to learn about one another's real needs. Community-driven medical education, research, and practice can expand our notions of transgender health, yielding rich relevant data, and ultimately higher quality care.

## Notes

^*^ The other study is Cumming R, Sylvester K, Fuld J. P257 Understanding the effects on lung function of chest binder use in the transgender population. Thorax. 2016;71:A227.

^†^ Transgender Health AMA Series: I'm Joshua Safer. Reddit, July 24, 2017. Available at: https://www.reddit.com/r/science/comments/6p7uhb/transgender_health_ama_series_im_joshua_safer

^‡^ McNamara B. Experts Answer Reddit Questions About Transgender People. Teen Vogue, July 28, 2007. Available at: https://www.teenvogue.com/story/reddit-transgender-ama-busts-myths

^§^ “Transgender Health: Patients & Providers.” Available at: https://genderqueer.me/transgender-patients-providers

^**^ *Genderqueer.me* has over 20,000 followers across social platforms.

^††^ “Approach to genderqueer, gender non-conforming, and gender nonbinary people.” Available at: http://transhealth.ucsf.edu/trans?page=guidelines-gender-nonconforming

^‡‡^ “Top 5 Questions On Hormones.” Available at: https://genderqueer.me/2017/10/13/fv-top-5-questions-about-hormones

^§§^ “Expect Excellence.” Available at: https://genderqueer.me/2017/06/13/fv-expect-excellence/#comment-32394
